# *SlMAPK3* enhances tolerance to tomato yellow leaf curl virus (TYLCV) by regulating salicylic acid and jasmonic acid signaling in tomato (*Solanum lycopersicum*)

**DOI:** 10.1371/journal.pone.0172466

**Published:** 2017-02-21

**Authors:** Yunzhou Li, Lei Qin, Jingjing Zhao, Tayeb Muhammad, Hehe Cao, Hailiang Li, Yan Zhang, Yan Liang

**Affiliations:** 1 College of Horticulture, Northwest A&F University, Yangling, Shaanxi, P. R. China; 2 College of Plant Protection, Northwest A&F University, Yangling, Shaanxi, P. R. China; 3 College of Natural Resources and Environment, Northwest A&F University, Yangling, Shaanxi, P. R. China; Stony Brook University, UNITED STATES

## Abstract

Several recent studies have reported on the role of mitogen-activated protein kinase (MAPK3) in plant immune responses. However, little is known about how MAPK3 functions in tomato (*Solanum lycopersicum* L.) infected with tomato yellow leaf curl virus (TYLCV). There is also uncertainty about the connection between plant MAPK3 and the salicylic acid (SA) and jasmonic acid (JA) defense-signaling pathways. The results of this study indicated that *SlMAPK3* participates in the antiviral response against TYLCV. Tomato seedlings were inoculated with TYLCV to investigate the possible roles of *SlMAPK1*, *SlMAPK2*, and *SlMAPK3* against this virus. Inoculation with TYLCV strongly induced the expression and the activity of all three genes. Silencing of *SlMAPK1*, *SlMAPK2*, and *SlMAPK3* reduced tolerance to TYLCV, increased leaf H_2_O_2_ concentrations, and attenuated expression of defense-related genes after TYLCV infection, especially in *SlMAPK3*-silenced plants. Exogenous SA and methyl jasmonic acid (MeJA) both significantly induced *SlMAPK3* expression in tomato leaves. Over-expression of *SlMAPK3* increased the transcript levels of SA/JA-mediated defense-related genes (*PR1*, *PR1b*/*SlLapA*, *SlPI-I*, and *SlPI-II*) and enhanced tolerance to TYLCV. After TYLCV inoculation, the leaves of *SlMAPK3* over-expressed plants compared with wild type plants showed less H_2_O_2_ accumulation and greater superoxide dismutase (SOD), peroxidase (POD), catalase (CAT), and ascorbate peroxidase (APX) activity. Overall, the results suggested that *SlMAPK3* participates in the antiviral response of tomato to TYLCV, and that this process may be through either the SA or JA defense-signaling pathways.

## Introduction

Plants are threatened by a variety of abiotic and biotic stresses. Among biotic stresses, viruses are the most serious pathogens. Plants have evolved sophisticated mechanisms to defend against viral infection by limiting virus replication and movement [1]. These mechanisms include resistance that is either induced or mediated by salicylic acid (SA), jasmonic acid (JA)-, and RNA interference (RNAi). All of these mechanisms play important roles in plant antiviral defense systems [2–8].

If they survive an initial pathogen attack, plants can exhibit enhanced resistance to subsequent infection by a broad range of pathogens. This induced resistance, which requires the endogenous plant hormone SA, is known as systemic acquired resistance (SAR) [9,10]. Exogenous application of certain natural or synthetic compounds [e.g., SA and Me (methyl) JA] can also induce resistance [11, 12]. In plants, induced resistance is often associated with “cell priming” [13, 14]. Priming enables cells to respond to less stimulus in a rapid and robust manner [15–17]. Priming is thought to be the basis of induced resistance to all plant pathogens [16, 18]. Induced resistance can cause faster and stronger activation of defense responses when plants experience either biotic or abiotic stress [15]. A previous report indicated that MAPK3 and MAPK6 are critical for full priming of stress responses in *Arabidopsis* [19]. Previous studies have shown that MAPKs have an important role in defense against pathogens [20–22].

Tomato yellow leaf curl virus (TYLCV), a Begomovirus genus within the *Geminiviridae*, causes tomato yellow leaf curl disease (TYLCD), one of the most devastating diseases affecting tomato (*Solanum lycopersicum* L.) production in recent decades [23–25]. The virus is found throughout the world and is still spreading in China and other countries [25, 26]. Infection by TYLCV leads not only to leaf stunting, yellowing, and curling but also to flower abortion. The TYLCV is mainly transmitted leaf-to-leaf by whitefly *(Bemisia tabaci* Gennadius*)*. Recent studies suggest that TYLCV is also seed-transmissible [27]. Resistance to TYLCV has been observed in wild type tomato (e.g., *S*. *chmielewski*, *S*. *chilense*, *S*. *pimpinellifolium*, *S*. *habrochaites*, *S*. *glandulosum*, *S*. *peruvianum* and *S*. *lycopersicoides*). Five resistance/tolerance genes (*Ty-1*, *Ty-2*, *Ty-3*, *Ty-4* and *Ty-5*) to TYLCV have been mapped and identified [28–36]. Some resistance markers have been used in introgression breeding. Interestingly, TYLCV can still be detected in plants that have these resistance genes [37]. Recent cloning experiments suggest that *Ty-1* and *Ty-3* are allelic [34, 35]. *Ty-1* and *Ty-3* both encode RNA-dependent RNA polymerase (RDR). It has been hypothesized that *Ty-1* and *Ty-3* and may be involved in RNA silencing [34, 35] by increasing cytosine methylation of viral genomes [38].

Transgenic methods offer great potential for enhancing the internal defense mechanisms of plants against viruses. However, consumers have not widely accepted genetically modified organisms (GMOs) because of concern about the effect of GMOs on human health. Recently, SA and JA were used in a nontransgenic approach to inhibit RNA viruses in tomato (*Lycopersicon esculentum*), hot pepper (*Capsicum frutescens*), and tobacco (*Nicotiana benthamiana*) [39]. It was reported that SA and JA inhibited not only virus replication but also cell-to-cell and long-distance movement of the virus [39, 40]. One hypothesis is that SA and JA enhance plant resistance by triggering either induced resistance or SAR [18]. This kind of resistance needs the participation of MAPK [41–43].

Mitogen-activated protein kinase (MAPK) cascades are three-tiered signaling kinase modules [44, 45]. Their main function is to transmit extracellular stimuli into intracellular responses [44]. MAPK cascades also amplify the primary signal [44, 45]. It has been suggested that MAPK is connected with or dependent on JA and SA signaling [41–43]. Several studies have reported on the role of MAPK3 in plant stress responses [19, 46–48]. More importantly, *AtMAPK3* is required for fully priming of stress responses in *Arabidopsis* [19]. However, little is known about the function of MAPK3 in antiviral activity against compatible viruses such as TYLCV. MPK3 has been shown to play a pivotal role in SA- and JA-mediated defense in *Arabidopsis* and *japonica* rice [49, 50]. More importantly, previous studies indicated that JA and/or SA can enhance plant defense against RNA viruses [39]. Several studies suggest that SA or JA-triggered resistance against either biotic or abiotic stress may be associated with MAPK signaling cascades [47, 48, 51–53]. There is still uncertainty about the relationship between plant MAPKs and the SA- and JA- defense signal pathways in regard to antiviral activity. The objectives of this study were (i) to analyze the function of *MAPK3* in the antiviral defense response of tomato to TYLCV and (ii) to learn more about the relationship between MAPK3 and the SA- and JA- defense signal pathways.

## Materials and methods

### Plant materials and growth conditions

**Fig 1:**
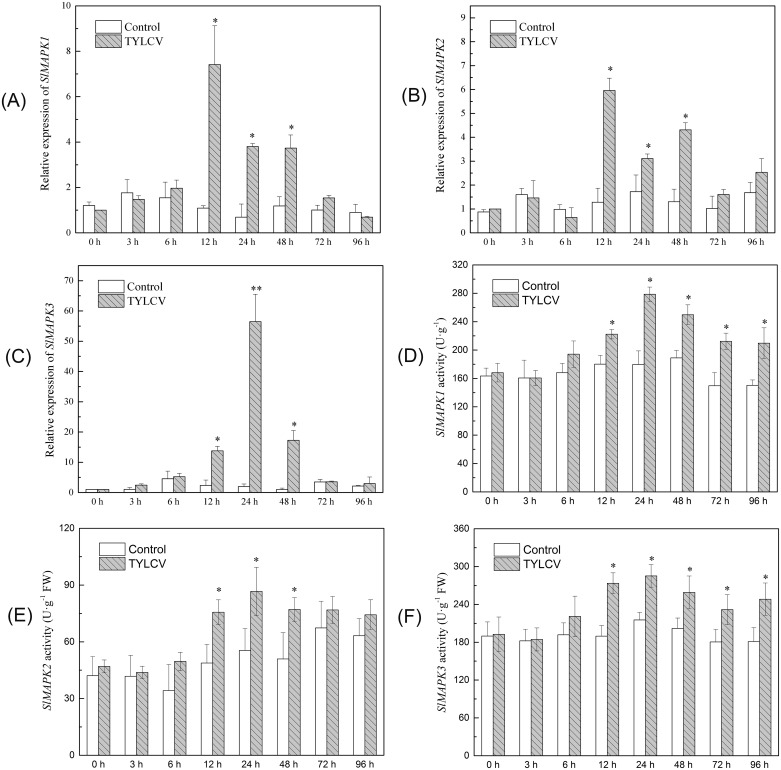
The RNA expression and protein activity of *SlMAPK1*, *SlMAPK2 and SlMAPK3* were analyzed in ‘Y19’ tomato leaves following TYLCV infection. Samples were collected from the uppermost leaves at different times after TYLCV inoculation. The samples were used to extract RNA, which was reverse transcripted into cDNA to detect the relative expression of *SlMAPK1* (A), *SlMAPK2* (B) and *SlMAPK3* (C) by qRT-PCR. The tomato *SlEF1α* gene was used as an internal control [59, 64]. The expression levels are relative to 0 h post infection. The activities of *SlMAPK1* (D), *SlMAPK2* (E) and *SlMAPK3* (F) were determined with an ELISA kit (Shanghai Biological Technology Co., Ltd.). Values are means ± standard error (SE), replicated thrice. The treatments were compared with the control using Tukey’s test. * Significant at P<0.05, ** Significant at P<0.01.

**Fig 2:**
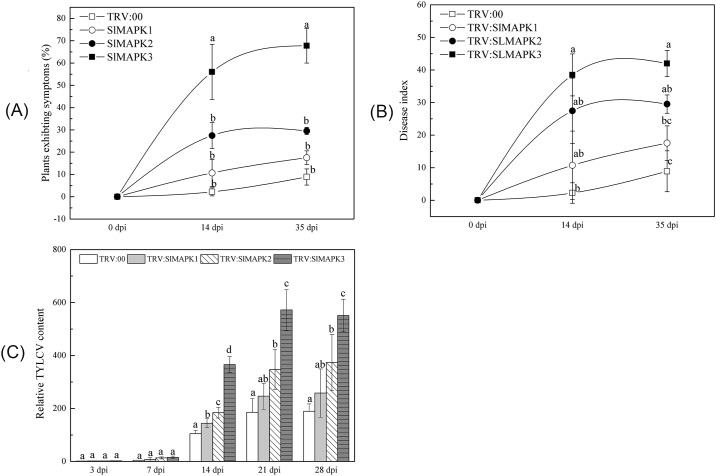
*SlMAPK3*-silencing reduced the tolerance of ‘Y19’ tomato to TYLCV. (A) The percentage of *SlMAPK*-silenced (TRV: *SlMAPK1*-, TRV: *SlMAPK2*-, and TRV: *SlMAPK3*) and non-silenced (TRV:00-, control) plants exhibiting TYLCV symptoms at 0, 14, and 35 dpi. Each treatment had 15–20 ‘Y19’ plants, replicated thrice. (B) Disease index of TYLCV in *SlMAPK*-silenced (TRV: *SlMAPK1*-, TRV: *SlMAPK2*-, and TRV: *SlMAPK3*) and non-silenced (TRV: 00-, control) plants at 0, 14, and 35 dpi. Each treatment had 15–20 ‘Y19’ plants, replicated thrice. (C) Relative TYLCV content in *SlMAPK*-silenced (TRV: *SlMAPK1*-, TRV: *SlMAPK2*-, and TRV: *SlMAPK3*) and non-silenced (TRV: 00-, control) plants at 3, 7, 14, 21, and 28 dpi. Leaf samples were collected from all plants, whether or not they displayed symptoms. The leaf samples were mixed within a treatment and then analyzed to determine total DNA. Three biological replicates were performed. The relative TYLCV content in the samples was determined using qPCR. The results are means ± standard error (SE), replicated thrice. The treatments were compared with the control using Tukey’s test. Different letters indicate significant differences at P<0.05.

**Fig 3:**
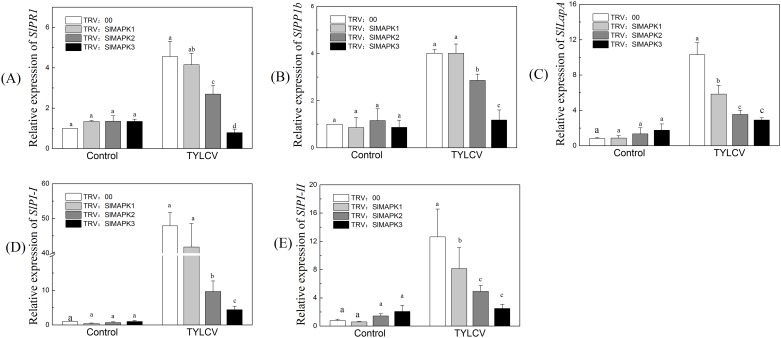
Defense-related gene expression decreased when *SlMAPK1*, *SlMAPK2*, *SlMAPK*3-silenced ‘Y19’ tomato plants were infected with TYLCV. (A-E) The relative expression of the SA-mediated defense marker genes *SlPR1* (A), *SlPR1b* (B) and the JA-mediated defense marker genes *SlLapA* (C), *SlPI-I* (D) and *SlPI-II* (E) at 14 dpi with TYLCV. Values are means ± standard error (SE), replicated thrice. The treatments were compared with the control using Tukey’s test. Different letters indicate significant differences at P<0.05.

**Fig 4:**
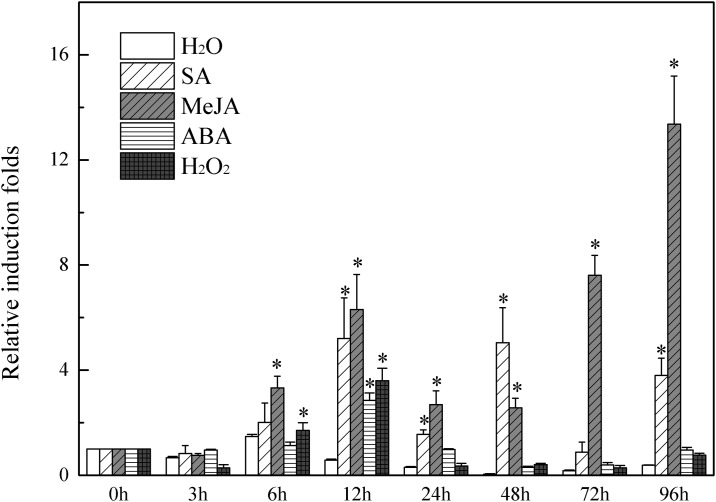
Exogenous application of signaling molecules increased *SlMAPK3* expression in ‘Y19’ tomato leaves. Tomato seedlings were treated with either 100 μM SA, 100 mM MeJA, 10 mM H_2_O_2_, 100 μM ABA, or water (i.e. mock treatment). The tomato *SlEF1α* gene was used as an internal control [59, 64]. Values are means ± standard error (SE), replicated thrice. The expression levels are relative to 0 h. The treatments were compared with the control using Tukey’s test. * Significant at P<0.05.

**Fig 5:**
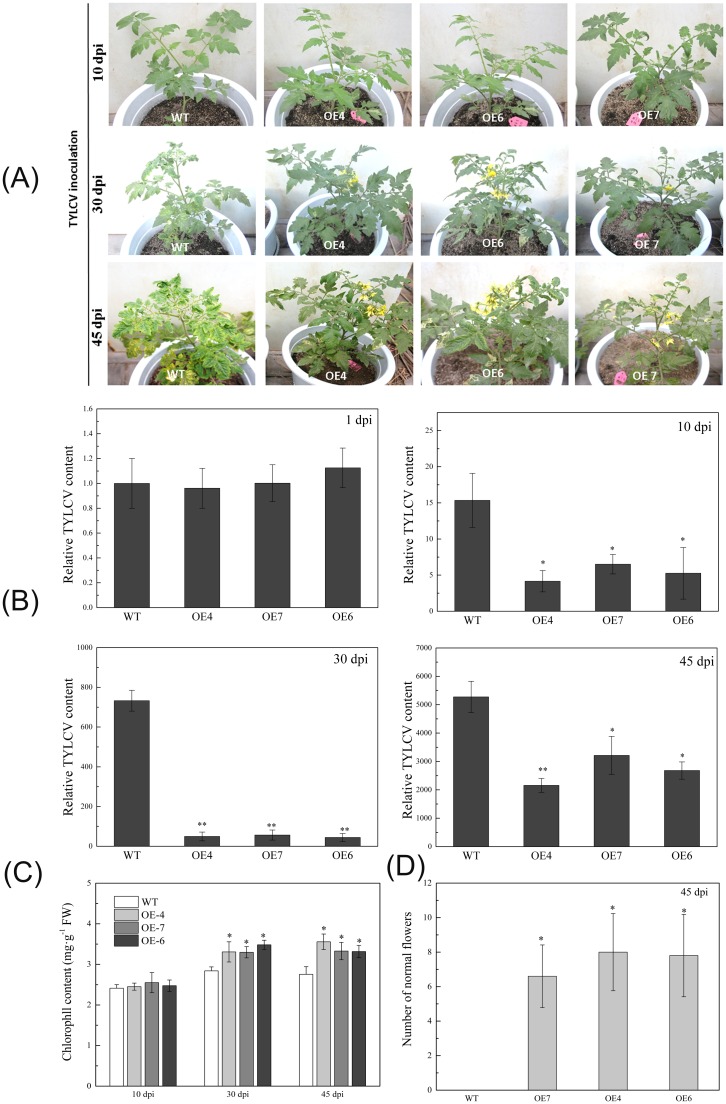
Overexpression of *SlMAPK3* in ‘M82’ tomato enhanced tolerance to TYLCV. (A) Phenotype analysis of wild type (WT, ‘M82’) and *SlMAPK3-*overexpressed (OE-4, OE-6 and OE-7) plants at 10, 30, and 45 dpi with TYLCV. (B) The relative TYLCV content in WT and *SlMAPK3-*overexpressed lines at 1, 10, 30, and 45 dpi. Values are means ± standard error (SE), replicated thrice. Tomato *β-actin* was used as an internal control for qRT-PCR [62, 63]. The expression levels are relative to 1 dpi. (C) Leaf chlorophyll contents in WT and *SlMAPK3-*overexpressed lines. Values in B-D are means ± SE of at least three replicates. (D) The average number of flowers on WT and *SlMAPK3-*overexpressed plants at 45 dpi. Values are means ± SE of at least three replicates. Significant differences between the OE lines and the WT lines were compared using Tukey’s test. * Significant at P<0.05, ** Significant at P<0.01.

**Fig 6:**
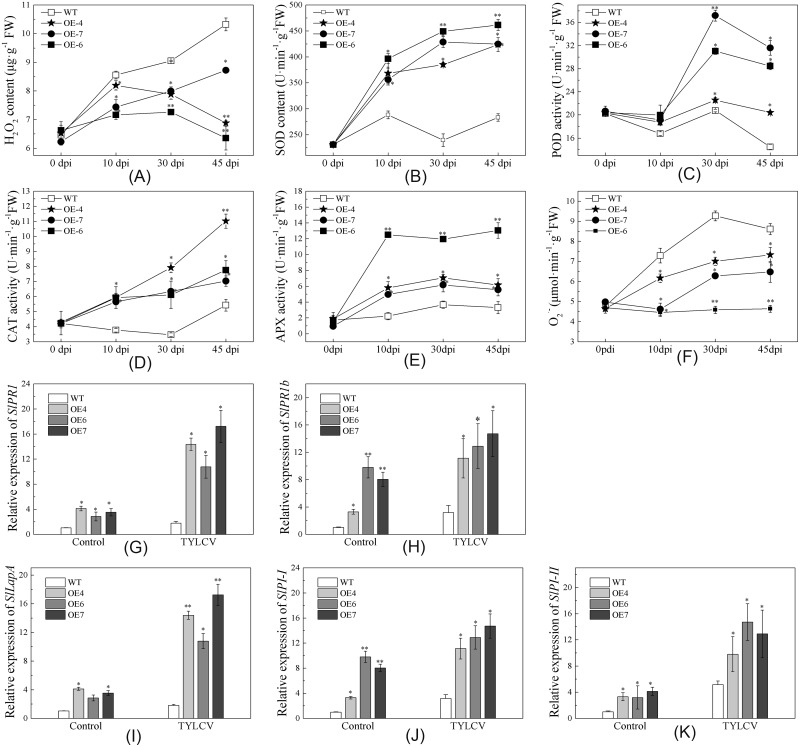
H_2_O_2_ accumulation, antioxidant enzyme activity, and defense-related gene expression in wild type (WT) and *SlMAPK3-*overexpressed (OE4, OE6, OE 7) lines after infection with TYLCV. (A-F) H_2_O_2_ accumulation and antioxidant enzyme activity at 0, 10, 30, and 45 dpi. The second and third uppermost leaves were collected from each plant and analyzed for H_2_O_2_ content (A), SOD activity (B), POD activity (C), CAT activity (D), APX activity (E), and O_2_^-^ content (F). Each bar represents the mean of three replicates ± standard error (SE). (G-K) Expression analysis of defense-related genes, *SlPR1* (G), *SlPR1b* (H), *SlLapA* (I), *SlPI-I* (J) and *SlPI-II* (K) at 14 dpi with TYLCV. The *SlEF1α* gene was used as an internal control [59, 64]. Data are means ± SE (N = 3 to 9) of three independent experiments. Double asterisk (**) and single asterisk (*) indicate significant differences relative to controls at *P*< 0.01 (Tukey’s test) and *P*<0.05 (Tukey’s test), respectively.

Tomato line ‘Y19’ (with *Ty-1* and *Ty-3* markers, S1 Fig), three transgenic lines with overexpression of *SlMAPK3* (OE4, OE6, and OE7, Accession No. AY261514), and their wild-type (WT, ‘M82’) were used in this study. The three OE lines were from our lab and described previously [54]. The seeds were germinated on wet filter paper in Petri dishes in the dark at 28°C for 3 d. The seedlings were moved to a growth chamber with a 16 h light:8 h dark photoperiod and a 25/16°C temperature cycle. At the four true-leaf stage, the plants were transferred to a solar greenhouse near Northwest A&F University, Yangling, Shaanxi Province China. During the TYLCV inoculation and VIGS experiments, the plants were grown under a 22/18°C day/night temperature cycle. The results in Figs 1–4, S1, S3 and S4 Figs were obtained using tomato line Y19. The results in Figs 5 and 6 are for the OE lines (i.e., OE4, OE5, and OE6) and ‘M82’.

### TYLCV inoculation

The TYLCV infectious clone was provided by Professor Zhou Xueping of Zhejiang University [55]. The clone was introduced into *Agrobacterium* GV3101 and then injected into the phloem of 6-week-old plants as described previously [56]. The injections were done with a 1.0 mL syringe at three points (10 cm apart) on the stem. The first injection point was 10 cm above the soil surface. Plants infected with empty vectors were used as controls. Virus infection was determined visually and confirmed through PCR (S2 Fig) [57]. Each treatment had 15–20 plants. Three biological replicates were performed for these experiments.

### VIGS experiment

The pTRV1 and pTRV2 VIGS vectors were obtained from Dr. Dinesh-Kumar of Yale University [58]. Fragments of *SlMPK1*, *SlMPK2*, and *SlMPK3* were amplified using specific primers containing *Xho*I (5’ end) and *Sac*I (3’ end) sites and inserted into the pTRV2 vector. The TRV: *PDS* (phytoene desaturase) construct, which is used as a marker of VIGS silencing in plants, was made as described previously [59]. The constructs were introduced into *Agrobacterium* GV3101 by electroporation and injected into fully-expanded leaves of 3-week-old tomato plants according to Li et al. [59]. The primers used for construction of the vectors are listed in S1 Table. Silencing frequency (%) and silencing efficiency were calculated as described previously [59].

### Signaling molecules and hormonal treatment

Tomato plants at the 5-leaf stage were treated by foliar spraying with either 10 mM H_2_O_2_, 100 μM MeJA, 100 μM SA, 100 μM ABA, or water (i.e, mock spray) [60]. The top leaves of the plants were collected at 0, 3, 6, 12, 24, 48, 72, and 96 h. The leaves were immediately frozen in liquid N and then stored at -80°C for further analysis.

### DNA / RNA isolation and quantitative PCR (qPCR)

Systemic leaves were collected from three TYLCV-inoculated plants and three uninoculated ones. The leaves were immediately frozen in liquid N and kept at -80°C. Total DNA was extracted from the leaves using the cetyltrimethyl-ammonium bromide (CTAB) method [61]. Quantitative PCR (qPCR) was used to detect TYLCV in the total DNA samples as described previously [62]. The *β-actin* gene was used as a control for qPCR detection of TYLCV [62, 63]. Total RNA was isolated using an RNA extraction kit (Invitrogen, USA). The cDNA was synthesized using MultiScribe reverse transcriptase (Takara, China). Quantitative real-time RT-PCR (qRT-PCR) was performed using SYBR Premix Ex Taq II (Takara, China) on an iQ5 Real-Time PCR Detection System (BIO-RAD, USA). The expression of the *SlMAPK* genes and the defense-related genes was determined using qRT-PCR. The elongation factor 1-α (*SlEF1α*) gene was used as an internal reference [59, 64]. Three biological replicates were performed for these experiments. The gene specific primers for qRT-PCR are listed in S1 Table.

### Disease evaluation in transgenic plants

The percentage of plant exhibiting disease symptoms (%) and the disease index were determined at 14 and 35 days post inoculation (dpi) in the VIGS experiments. The calculation formulas of disease incidence (%) and disease index were as follows. Disease incidence = Number of plants with disease symptom/Number of all tested plants × 100%. Disease index = [∑ (Number of plants in a scale ×Corresponding scale value) / (Total number of plants × Highest scale value)] ×100.

Transgenic plants overexpressing *SlMAPK3* (OE4, OE6 and OE7) were inoculated with TYLCV as described above. The TYLCV contents were detected by qPCR [62]. Three biological replicates were performed for these experiments. The number of flowers was counted at 45 dpi. Disease severity was evaluated using a rating scale of 0 to 4, in which, 0 = no disease symptoms, 1 = slight symptoms visible only on close inspection, 2 = symptoms apparent at a distance of two-thirds of a meter from the plant, 3 = severe symptoms over the entire plant, and 4 = severe symptoms and stunting [65]. Intermediate scores (e.g., 0.5, 1.5, and 2.5) were used to allow more precise rating [30].

### Physiological parameters and enzyme activity

The activities of *SlMAPK1*, *SlMAPK2* and *SlMAPK3* were determined using an ELISA kit (Shanghai Biological Technology Co., Ltd.). Leaf H_2_O_2_ was measured by the method of Jiang and Zhang (2001) [66]. Leaf chlorophyll and superoxide O_2_^-^ were measured according to the methods of Porra et al. (1989) [67] and Wang and Luo (1990) [68], respectively. The activities of the antioxidant enzymes catalase (CAT), peroxidase (POD), acerbate peroxidase (APX), and superoxide dismutase (SOD) were assayed from leaves as described previously [69].

### Statistical analysis

Analysis of variance (ANOVA) was conducted using SPSS version 12.0 software. The significance of differences between means was determined by Tukey’s test. Data are presented as means ± standard error (SE). Double (**) and single (*) asterisks indicate significant differences relative to controls at P <0.01 and P <0.05, respectively. Different letters indicate significant differences compared to control at P <0.05.

## Results

### The RNA expression and protein activity of *SlMAPK1*, *SlMAPK2* and *SlMAPK3* was induced after infection with TYLCV

The first step in this experiment was to analyze changes in *SlMAPK* expression after TYLCV infection. A preliminary study indicated using PCR showed 100% inoculation success using this method (data not shown). *SlMAPK1*, *SlMAPK2*, and *SlMAPK3* expression was induced by TYLCV infection; however their expression levels were different (Fig 1A–1C). *SlMAPK1*, *SlMAPK2*, and *SlMAPK3* expression reached peaks between 12 and 24 h post infection and then declined. The expression of *SlMAPK1* and *SlMAPK2* in TYLCV infected plants was 7.4 and 5.9 fold greater at 12 h than at 0 h post inoculation. *SlMAPK3* expression was 56.5 fold greater at 24 h than at 0 h post inoculation. (Fig 1C).

The activities of *SlMAPK1*, *SlMAPK2*, and *SlMAPK3* were analyzed by ELISA at different times after TYLCV inoculation. *SlMAPK1*, *SlMAPK2*, and *SlMAPK3* were activated by TYLCV inoculation at 12 h after TYLCV inoculation (Fig 1). The activities of *SlMAPK1* and *SlMAPK3* were significantly greater than that of the control between 12 and 72 h after TYLCV inoculation. In comparison, *SlMAPK2* activity was greater than that of the control between 12 and 48 h after TYLCV inoculation. These results indicated that *SlMAPK1*, *SlMAPK2* and *SlMAPK3* all responded to TYLCV infection at both the RNA and protein levels; however, the expression and activity levels were different. Among the three, *SlMAPK3* had the strongest response to TYLCV infection.

### Silencing of *SlMAPK3* reduced tolerance to TYLCV

The second step of the experiment was to silence the *SlMAPK* genes by inoculating seedlings at the 3-leaf stage with TRV. The silencing frequency of VIGS technology was confirmed 12–40 d post infiltration with TRV: *SlPDS* (S3 Fig). Plants inoculated with TRV: *SlPDS* exhibited photo-bleaching two weeks after TYLCV inoculation. The VIGS-plants were inoculated with TYLCV 14 d later. The silencing efficiency and specificity were also checked (S4 Fig). This demonstrated that *SlMAPK1*, *SlMAPK2* and *SlMAPK3* had been silenced in TRV: *SlMAPK1*, TRV: *SlMAPK2* and TRV: *SlMAPK3*-infiltrated plants. The disease symptoms were evaluated at 0, 14, 35 dpi. The percentage of TRV: *SlMAPK3*-infiltrated plants exhibiting TYLCV symptoms increased to 56% at 14 dpi and 68% at 35 dpi (Fig 2A). These values were significantly greater than those of the control (TRV: 00). The percentage of TRV: *SlMAPK1* and TRV: *SlMAPK2*-infiltrated plants exhibiting symptoms was not significantly different from the control. The disease index of TRV: *SlMAPK3*-infiltrated plants was 38.5 at 14 dpi and 42.0 at 35 dpi (Fig 2B). These values were significantly greater than those of the control (2.2 at 14 dpi and 8.8 at 35 dpi).

The relative TYLCV content, which was determined by qPCR, increased with the development of disease (Fig 2C). The relative TYLCV contents of *SlMAPK2*- and *SlMAPK3*-silenced plants were significantly greater than those of the control at 14, 21, and 28 dpi. *SlMAPK3*-silenced plants had the highest relative TYLCV content in this study. It is interesting to note that the relative TYLCV content was significantly greater in *SlMAPK1*-silenced plants than in the control at 14 dpi. These data showed that *SlMAPK* silencing, particularly *SlMAPK3* silencing, reduced resistance and increased susceptibility to TYLCV. Thus, *SlMAPK3* might have an important role in regulating resistance against TYLCV in tomato.

### Silencing of *SlMAPKs* reduced defense-related gene expression

The third step in the experiment was to compare defense-related gene expression in *SlMAPK-*silenced and non-silenced plants. The SA- and JA- mediated signaling pathways regulate the expression of certain defense marker genes. Specifically, the SA-mediated pathway regulates *SlPR1* and *SlPR1b*. The JA-mediated signaling pathway regulates *SlLapA*, *SlPII*, and *SlPIII*. Therefore, the expression of these genes was analyzed to examine a possible molecular mechanism related to reduced TYLCV resistance in *SlMAPK1*-, *SlMAPK2*- and *SlMAPK3*-silenced plants. To do this, defense-related gene expression in TRV: *SlMAPK1*-, TRV: *SlMAPK2*- and TRV: *SlMAPK3*- silenced plants was compared with that in TRV-empty vector (TRV: 00)-infiltrated plants. *SlMAPK*-silencing had no effect on the activity of the SA- and JA-mediated defense genes (Fig 3). In contrast, *SlMAPK-*silenced plants with TYLCV inoculation exhibited significant differences in the expression of both *SlPRP1* and *SlPR1b* at 14 dpi. The expressions of *SlPRP1* and *SlPR1b* were greatest in TRV: 00 and least in TRV: *SlMAPK3* (Fig 3B and 3C). Similar patterns were observed for *SlLapA*, *SlPII*, and *SlPIII* expression (Fig 3D and 3F). Overall, *SlMAPK* silencing, especially TRV:*SlMAPK3* silencing, significantly reduced the expression of defense-related genes regulated by SA- and JA- mediated signaling pathways in TLYCV-infected plants.

### Exogenous application of various signalling molecules induced *SlMAPK3* expression

The fourth step in the experiment was to measure *SlMAPK3* expression in tomato leaves after exogenous application of various signaling molecules. As shown in Fig 4, all four types of signaling molecules significantly increased *SIMAPK3* expression at 12 h after application. Among the signaling molecules, exogenous MeJA had the most striking effect, increasing *SIMAPK3* expression at each sampling time between 6 and 96 h after application. *SlMAPK3* expression in the MeJA treatment reached a maximum 13.4 fold increase at 96 h. Exogenous SA significantly increased *SlMAPK3* expression at 12, 24, 48, and 96 h. The maximum increase (5.2 fold) was observed at 12 h. Exogenous ABA and H_2_O_2_ significantly increased *SlMAPK3* expression at 12 h by 3.6 and 2.9 fold, respectively. The results indicated that *SlMAPK3* responded significantly, but with different expression patterns, to exogenous MeJA and SA. This suggested that *SlMAPK3* could be involved in stress-activated signaling pathways regulated by SA and MeJA.

### *SlMAPK3* overexpression enhanced tolerance to TYLCV

To further confirm the role of *SlMAPK3* in antiviral defense, the TYLCV tolerance of three overexpression lines (i.e., OE4, OE6, and OE7) was compared with that of a WT line. No visible disease symptoms were observed on any of the plants at 10 dpi (Fig 5A). The WT line had typical TYLCV symptoms at 30 dpi, whereas the OE lines remained normal and developed flowers. At 45 dpi, the OE lines exhibited TYLCV symptoms but produced normal flowers. The WT line exhibited severe disease symptoms and was unable to produce normal flowers. Disease severity ratings reflected the patterns described above (Table 1). There was no difference in the disease ratings at 10 dpi. However, the ratings were significantly greater in the WT line than in the OE lines at 45 dpi.

**Table 1:** Disease severity rating in wild type (WT, ‘M82’) and *SlMAPK3-*overexpressed (OE-4, OE-6 and OE-7) lines at 10, 15, 30, and 45 dpi with TYLCV. Disease severity was rated using a scale from 0 (no disease) to 4 (severe symptoms and stunting).

	Disease severity rating			
	10 dpi	15 dpi	30 dpi	45 dpi
**WT**	0	1.3±0.6	2.33±0.28	3.33±0.58
**OE7**	0	0 **	0.33±0.58**	0.5±0.50**
**OE4**	0	0 **	0**	0.33±0.58**
**OE6**	0	0 **	0**	0.67±0.29 **

Plants of each genotype were agro-inoculated with the infectious TYLCV construct. Data represent means ± standard error (SE), n = 12–15. Significant differences between OE lines and WT were compared using Tukey’s test.

** Significant at P<0.01.

The relative TYLCV contents were the same in all lines at 10 dpi (Fig 5B). However, relative TYLCV contents were significantly less in the OE lines than in the WT line at 10, 30, and 45 dpi. At 45 dpi, relative TYLCV contents increased in the OE lines and produced visible symptoms (Fig 5B). One visible symptom of TYLCD is leaf yellowing due to a reduction in the number of chloroplasts per cell [70]. Leaf chlorophyll contents were significantly greater in the OE lines than in the WT line at 30 and 45 dpi (Fig 5C). The number of normal flowers was significantly greater in the OE lines than in the WT line at 45 dpi (Fig 5D). Overall, these results showed that the appearance of TYLCD symptoms was delayed in plants with *SlMAPK3* overexpression.

### *SlMAPK3* over-expression enhanced antioxidant capacity and defense-related gene expression

Previous studies have shown that biotic and abiotic stresses damage plants through accumulation of ROS during oxidative stress [71, 72]. In this experiment, there were no clear differences in H_2_O_2_ and O_2_^-^ accumulation among the WT and OE lines prior to TYLCV inoculation (i.e., 0 dpi). However, after inoculation, H_2_O_2_ and O_2_^-^ concentrations were both greater in the WT line than in the OE lines (Fig 6A and 6B). Plants have evolved complicated antioxidant defense systems to clear excess ROS and maintain cellular ROS homeostasis [71, 72, 73]. The enzymes CAT, SOD, APX, and POD are involved in the antioxidant defense system. There were no observable differences in enzyme activity between the WT and OE lines prior to TYLCV inoculation (Fig 6C–6F). However, the OE lines compared with the WT plants had significantly greater enzyme activity after TYLCV inoculation. These results suggested that *SlMAPK3* over-expression inhibited ROS production and had major influence on antioxidant capacity in transgenic plants.

The expression of five genes related to plant defense was also determined to increase insight into the molecular mechanisms underlying enhanced tolerance to TYLCV in the OE lines after inoculation. Without TYLCV inoculation, the relative expression of all five defense genes were greater in the OE lines than in the WT line (Fig 6). Inoculation with TYLCV significantly up-regulated the genes in all lines; the greatest increases were observed in the OE lines (Fig 6G–6K). This demonstrated that *SlMAPK3* over-expression enhanced the transcript levels of SA- and MeJA-mediated defense-related genes in both TYLCV inoculated and uninoculated plants.

## Discussion

*SlMAPK3* participated in an antiviral response to TYLCV and was induced by SA and JA hormones. VIGS-silencing of *SlMAPK3* increased viral infection compared with non-silenced plants. *SlMAPK3* silencing also reduced expression of defense-related genes in the SA- and JA-mediated pathways. Compared with WT plants, over-expression of *SlMAPK3* in transgenic plants enhanced the expression of defense-related genes in the SA- and JA- mediated pathways and increased tolerance to TYLCV. These results suggest that *MAPK3* participates in the defense response to TYLCV. We propose that the antiviral role of *MAPK3* could be attributed to induced resistance triggered by the SA and JA signal pathways.

Three MAPKs (*SlMAPK1*, *SlMAPK2*, and *SlMAPK3)* were differentially induced and activated after TYLCV inoculation. Among these, *SlMAPK3* had the highest expression and activity in the inoculated plants. MAPK cascades are readily activated during the plant response to avirulent pathogens or to pathogen-derived elicitors. Two MAPKs, WIPK and SIPK, are activated by virus (TMV) infection in tobacco [74]. In *Arabidopsis* leaves, MAPKs are rapidly activated by biotic stresses [75, 76]. Similarly *LeMPK3* is specifically induced and activated in resistant tomato plants infected by avirulent strains of the phytopathogenic bacteria *Pseudomonas syringae* pv. tomato and *Xanthomonas campestris* pv. *vesicatoria* [77]. Interestingly, those MAPKs also participate in the response of several plant species to abiotic stresses [59, 60, 77–80]. The possibility exists that *MAPK3* acts as an upstream signaling kinase which regulates the response to abiotic and biotic stresses such as salt, drought, cold, and pathogens. [46–49, 59, 60,77–81]. These results support the understanding that MAPK3 cascades participate in the immune response of plants [47, 82, 83].

In this study, the genes downstream of the MAPKs changed with the down-regulation of MAPK in silenced plants (Fig 3). This agrees with a previous report that *PI-II* and *PI-I* were down-regulated in SpMPK3-silenced plants [84]. *SlMAPK1*-, *SlMAPK2*- and *SlMAPK3*-silenced plants all exhibited increased TYLCV content and reduced tolerance to TYLCV; however, *SlMAPK3*-silenced plants had the greatest disease incidence (Fig 2). These results were consistent with a previous study in which suppression of both *MPK3* and *MPK6* in transgenic *Arabidopsis* resulted in significant decreases in the induction of defense-related gene expression and pathogen resistance compared with wild-type plants [82]. Disease development and wilting symptoms of *Ralstonia solanacearum* also appeared more often in MPK3 silenced plants [81]. The VIGS-silencing experiment implied that the role of *SlMAPK3* in the defense response was somewhat different from that of *SlMAPK1* and *SlMAPK2*. This agrees with a previous report which showed that *MPK3* functioned differently than *MPK1* and *MPK2* in the response of tomato to wounds caused by *Manduca sexta* (*Lepidoptera*) [83]. These results suggested that although they belong to the same gene family, the response of *SlMAPK3* to TYLCV was different from that of *SlMAPK1* and *SlMAPK2*.

Tomato is infected with TYLCV by viruliferous whiteflies. Many factors (e.g., whitefly gender and development stage) influence the efficacy and stability of the inoculation. For this reason, we used an artificial system of TYLCV infection by agroinfiltration in this study. It should be noted that TYLCV transmission by whiteflies is circular and persistent [85]. Prolonged infection may result in a different response compared with our study. Previous research indicated that the abundance of mammalian extracellular signal-regulated kinase (ERK)-like protein (representing MAPKs) remained high for 1 d and then decreased slowly for 40 d after whitefly infestation [86]. This suggested that (i) ERK-like proteins negatively regulate the defense response or (ii) TYLCV can suppress the activity of the ERK-like protein after 1 dpi. Additional research needs to be done to determine SlMAPKs expression patterns in tomato inoculated by whiteflies. If there is decrease in SlMAPKs levels during prolonged infection, it would suggest that the MAPK response to stress was complete within a short time after infection.

Upstream signaling components such as ROS, auxin, abscisic acid, and phosphatidic acid have been reported to be involved in MAPK activation [87]. In our results, the WT line compared with the OE lines accumulated more H_2_O_2_, leading to decreases in total leaf chlorophyll (Fig 5C). The ROS can not only induce hypersensitive response (HR) due to damage after pathogen infection, but also inhibit the spread of cell death to neighboring cells by programmed cell death. Previous studies have reported that TYLCV infection does not induce HR in normal plants [88]. It is possible that the immune system becomes weaker in *SlMAPK3*-silenced plants, leading to HR-like lesions. Similarly, Faoro and Gozzo reported in their review that compatible viruses can also cause systemic necrosis leading to plant death [40, 89]. In addition, the activity of the antioxidant enzyme APX was enhanced after TYLCV inoculation in *SlMAPK3-* overexpressed plants compared with WT plants (Fig 6E). Recent studies reported that TYLCV down regulated *APX1/2* transcription through mitigation of its regulator heat shock transcription factors *HSFA2* under a combination of virus and heat stresses [90]. It is possible that the antiviral response of plants to a single stress is different than that to a combination stresses.

In plant immune systems, MPK3/MPK6 activation and rapid ROS burst are two independent, early signaling events [91]. Accumulation of H_2_O_2_ could lead to SA synthesis [92]; however TYLCV also enhanced SA accumulation during the early stages of infection [62]. High levels of SA along with H_2_O_2_ could activate local PR gene expression [93]. In effector-triggered immunity (ETI), the MPK3 and SA signaling pathways compensate each other regarding PR1 expression and pathogen inhibition [94]. Similarly, VIGS of *MPK1*, *MPK2*, and *MPK3* in tomato resulted in reductions in JA-mediated defense gene expression and defense responses [84]. We observed that the expression of SA- and JA- mediated defense-related genes was reduced in silenced plants after TYLCV inoculation. The largest decreases were in *SlMAPK3-*silenced plants (Fig 3). Exogenous SA and JA application induced *SlMAPK3* expression. This suggested that MAPK3 was linked with both pathways; however, it may be that MAPK3 had a role in balancing or reducing antagonism between SA and JA signaling. Modulated interactions between SA and JA may contribute significantly to induced resistance [95]. Molecular characterization of *MAPK3* in *Nicotiana attenuate* demonstrated that *WIPK* and *SIPK* were orthologs of *AtMPK3* and *AtMPK6*, respectively, and were involved in JA and SA signaling pathways and biosynthesis [76]. However, *OsMPK3* transcripts increased after treatment with JA but not SA [50].

In *Arabidopsis*, *AtMPK3* and *AtMPK6* positively regulated SA signaling [96]. After stress, phosphorylated active *AtMPK3* and *AtMPK6* were both correlated with enhanced expression of the *PR-1* gene [19]. Over-expression of a tobacco MPK3 homolog (WlPK) resulted in enhanced JA levels and expression of the JA responsive gene *P1-II* [97]. Similarly, *AhMPK3* over-expression increased *PI-II* expression, the amount of a basic pathogenesis-related protein, and plant resistance [98]. We observed that *SlMAPK3* transgenic plants compared with WT plants had greater expression of SA- and JA- defense-related genes and less TYLCV content. The reduction of TYLCV in *SlMAPK3* over-expressed plants may be due to increases in either the accumulation of SA and JA or the expression of defense-related genes in both pathways. Exogenous application of both SA and JA induced stronger resistance in tobacco against virus attack compared with application of SA or JA alone [39]. Previous reports indicate that SA not only has significant roles in the RNA silencing mechanism but also delays accumulation of RNA pathogens [99], perhaps due to pre-induction of RNA silencing-related genes by SA or GA [100]. The SA could act as an enhancer of RNA-silencing antiviral defense. An SA-mediated defense mechanism and an RNA-silencing mechanism acted together to reduce plum pox virus (PPV) infection in tobacco [101]. Based on these previous reports as well as our own findings, we propose that (i) MAPK3 could be an essential component in inducing “priming” of cells in virus-infected plants and (ii) MAPK3 plays an important role in the development of induced resistance against viruses by coordinating the expression of defense genes in SA and JA-mediated pathways. It should be noted that RNA silencing is thought to be an important antiviral defense mechanism. There is a possibility that *SlMAPK3* also acts as an upstream signaling kinase, triggering the RNA silencing pathway against TYLCV. Further in-depth study is required to test this hypothesis.

## Conclusions

*SlMAPK* (*SlMAPK1*, *SlMAPK2* and *SlMAPK3*) transcription and activity in tomato leaves was strongly induced by TYLCV infection, with *SlMAPK3* having the highest expression and activity among the three genes. Functional analyses by VIGS and overexpression showed that *SlMAPK3* may participate in regulating the defense response against TYLCV by modulating SA and JA- mediated defense responses in tomato.

## Supporting information

S1 Table List of primers used in this study. (A) Primer sequences used for qRT-PCR analysis.
(B) Primer sequences used for VIGS of *SlMAPK* genes in tomato.
(C) Primer sequences used for semi-qPCR to detect TYLCV in tomato
(D) Primer sequences used for cloning *SlMAPKs* in tomato
(DOCX)

S1 Fig Detection of the resistance markers (*Ty-1* and *Ty-3*) in ‘Y19’ and wild type ‘M82’ tomato. DNA was extracted from the top leaves of ‘Y19’and ‘M82’ plants. The resistance markers, *Ty-1* and *Ty-3*, were detected by PCR. The wild type ‘M82’ was used as a negative control.
(TIF)

S2 Fig TYLCV inoculation success in ‘Y19’, ‘M82’ and OE lines at 7 and 14 dpi. DNA was extracted from the top leaves of *SlMAPK*-silenced (TRV: *SlMAPK1*-, TRV: *SlMAPK2*-, and TRV: *SlMAPK3*-) plants, non-silenced (TRV: 00-, control) plants, ‘M82’ plants and OE plants at 7 and 14 dpi. PCR was used to confirm the success of artificial inoculation with TYLCV.
(TIF)

S3 FigSilencing of tomato gene which acts as an indicator for the frequency (%) of VIGS silencing.The silencing frequency (%) of VIGS technology (A) was calculated in TRV: *SlPDS* -infiltrated plants at 12, 15, 20, 25 30, 35 and 40 dpi. The following equation was used:
$\frac{\text{Number of plants showing silencing phenotype }\left(\text{bleaching or yellowing}\right)}{\text{Total number of plants infiltrated}}\times 100\%$Plants were infected with TRV vector carrying the phytoene desaturase (*PDS)* gene of tomato. Silencing of the endogenous *PDS* results in the inhibition of carotenoid biosynthesis, leading to a photo-bleaching phenotype (B). Photographs taken 4 weeks after TRV infiltration.</SI_Caption>(TIF)

S4 Fig Silencing efficiency for *SlMAPK1*, *SlMAPK2* and *SlMAPK3*. The silencing efficiency was determined by qRT-PCR and compared with the control (defined as 100%). Values are the mean ± standard error (SE), replicated thrice. The treatments were compared with the control using Tukey’s test, * Significant at P<0.05.
(TIF)
