# Supplementary material for: SlMAPK3 enhances tolerance to tomato yellow leaf curl virus (TYLCV) by regulating salicylic acid and jasmonic acid signaling in tomato (Solanum lycopersicum)
Source: PLoS One. 2017 Feb 21;12(2):e0172466. doi: 10.1371/journal.pone.0172466 (PMC5319765; doi:10.1371/journal.pone.0172466)
Supplement: S1 Table — (A) Primer sequences used for qRT-PCR analysis. (B) Primer sequences used for VIGS of SlMAPK genes in tomato. (C) Primer sequences used for semi-qPCR to detect TYLCV in tomato (D) Primer sequences used for cloning SlMAPKs in tomato (DOCX) [file pone.0172466.s001.docx]

**Supplemental Table 1 Primers used in the study.**

**A. Primer sequences used for the quantitative RT-PCR analysis of gene-expression in tomato and relative content of TYLCV**

| **No.** | ***Name*** | **Primers sequence** | **Reference** |
| --- | --- | --- | --- |
| **1** | ***SlMAPK1*(**AY261512**)** | ATCCCAGAAGGAGAATAACAG | [59] |
|  |  | ATCAAACCTGcAACAATCTG |  |
| **2** | ***SLMAPK2*(**AY261513**)** | ATTCCACCACCTCAACGA | [59] |
|  |  | TGCTAGGCTTCAAGTCCC |  |
| **3** | ***SlMAPK3*(**AK319930**)** | GCAACTCCCACAACATCC | [59] |
|  |  | TCTGCTCTTCTCCTATCCCT |  |
| **4** | ***SlEF1α*(**X14449**)** | GACAGGCGTTCAGGTAAGG | [59] |
|  |  | CCAATGGAGGGTATTCAGC |  |
| **5** | ***PDS*(**NM_001247166.1**)** | GGCGCGAGCTCGGCACTCAACTTTATAAACC | [59] |
|  |  | CGGCGCTCGAGCTTCAGTTTTCTGTCAAACC |  |
| **6** | ***TYLCV-V1*(**AB110218**)** | GAAGCGACCAGGCGATATAA | [57,62] |
|  |  | GGAACATCAGGGCTTCGATA |  |
| **7** | ***SlPR1b*(**NM_001247385**)** | TTTCCCTTTTGATGTTGCT | [102] |
|  |  | TGGAAACAAGAAGATGCAGT |  |
| **8** | ***SlPR1a*(**NM_001247199**)** | AACGCTCACAATGCAGCTCGT | [102] |
|  |  | AAGGTCCACCAGAGTGTTGC |  |
| **9** | ***SIPII*(**NM_001308941**)** | GTTGTACAAATGCCTGTGGTGAC | [102] |
|  |  | GGTAAGAGTACATGAAGAGATGC |  |
| **10** | ***SlPIII* (**K03291**)** | CATCTTCTGGATTGCCCA | [102] |
|  |  | ACACACAACTTGATGCCCAC |  |
| **11** | ***SlLapA*(**NM_001246933**)** | GGGACTAATGATGTTTGGAA | [102] |
|  |  | GTGGCAATTTTATTTAGGCA |  |
| **12** | ***β-actin* (**BT013524**)** | GGAAAAGCTTGCCTATGTGG | [62] |
|  |  | CCTGCAGCTTCCATACCAAT |  |

**B. Primer sequences used for VIGS of SlMAPK1-3 genes in tomato**

| **No.** | **Gene name** | **Primers** | **Reference** |
| --- | --- | --- | --- |
| **1** | ***PDS*(XM_010320112)** | GGCGCGAGCTCGGCACTCAACTTTATAAACC | [59] |
|  |  | CGGCGCTCGAGCTTCAGTTTTCTGTCAAACC |  |
| **2** | ***SlMAPK1*(AY261512)** | ATAATTGCTGACAGATTGTTGC | [59] |
|  |  | CGGCGCTCGAGCATTTCAGTCTAAAATAAAATCCAC |  |
| **3** | ***SlMAPK2*(AY261513)** | GTACTCGCTCGTTTGCTGTT | [59] |
|  |  | CGGCGCTCGAGCATTTCTGGAACTAAAAATACAGAT |  |
| **4** | ***SlMAPK3* (AK319930)** | GCATAAGAGAAATCAGTTCTTCTCT | [59] |
|  |  | CGGCGCTCGAGACACCCAAAACTTCAAAATGAC |  |

**C. Primer sequences used for semi-quantitative PCR to detect TYLCV in tomato**

| **No.** | **Gene name** | **Primers** | **Reference** |
| --- | --- | --- | --- |
| **1** | ***TY-F/R*(530-928)** | ATTGGGCTGTTTCCATAGGGC | [57] |
|  |  | CACACGGATGGGAAATACTT | |
| **2** | ***Actin*(TC198350)** | GGAAAAGCTTGCCTATGTGG | [57] |
|  |  | CCTGCAGCTTCCATACC |  |

**D. Primer sequences used for cloning genes of SlMAPK1-3 in tomato**

| **No.** | **Gene name** | **Primers** | **Reference** |  |
| --- | --- | --- | --- | --- |
| **1** | ***SlMAPK1*(**AY261512**)** | CGGGATCCTATGGATGGTTCCGTTCC | [59] |  |
|  |  | GGCGCGAGCTCTCACATGCGCTGGTATTC |  |  |
| **2** | ***SLMAPK2*(**AY261513**)** | CGGGATCCTGAAATAGGAGAAAAGAGGGAAA | [59] |  |
|  |  | GGCGCGAGCTCTCACATGTGCTGGTATTCGG |  |  |
| **3** | ***SlMAPK3*(**AY261514**)** | CGGGATCCATGGTTGATGCTAATATGGGTG | [59] |  |
|  |  | GGCGCGAGCTCTTAAGCATATTCAGGATTCAACG | |  |

**Reference:**

102. Li XH, Zhang YF, Huang L, Ouyang ZG, Hong YB, Zhang HJ, et al. Tomato SlMKK2 and SlMKK4 contribute to disease resistance against Botrytis cinerea. Bmc Plant Biology. 2014;14. PubMed PMID: WOS:000338166100001.
