# Supplementary figures and images for: SlMAPK3 enhances tolerance to tomato yellow leaf curl virus (TYLCV) by regulating salicylic acid and jasmonic acid signaling in tomato (Solanum lycopersicum)
Source: PLoS One. 2017 Feb 21;12(2):e0172466. doi: 10.1371/journal.pone.0172466 (PMC5319765; doi:10.1371/journal.pone.0172466)

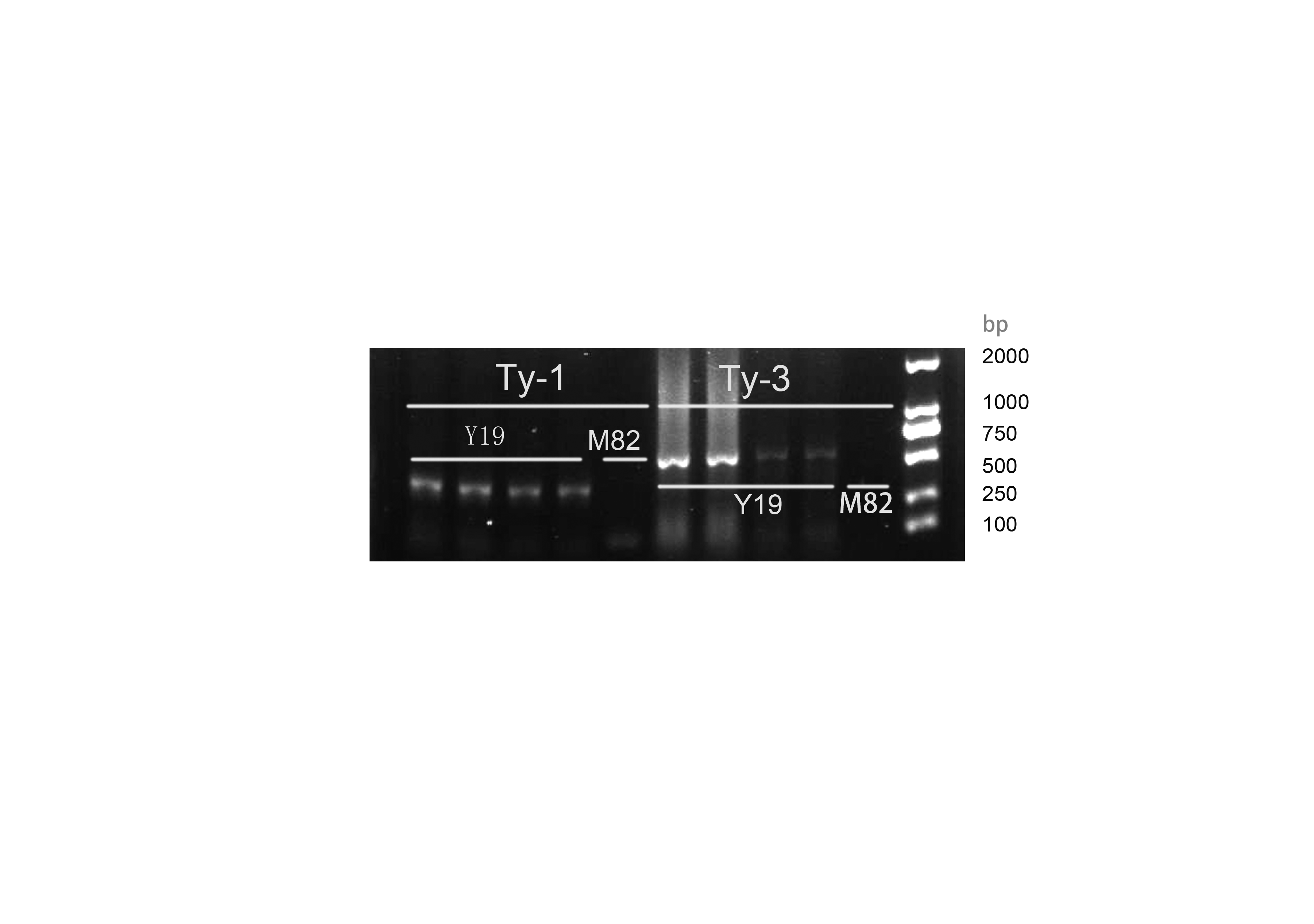

Supplement: S1 Fig — DNA was extracted from the top leaves of ‘Y19’and ‘M82’ plants. The resistance markers, Ty-1 and Ty-3, were detected by PCR. The wild type ‘M82’ was used as a negative control. (TIF) [file pone.0172466.s002.tif]

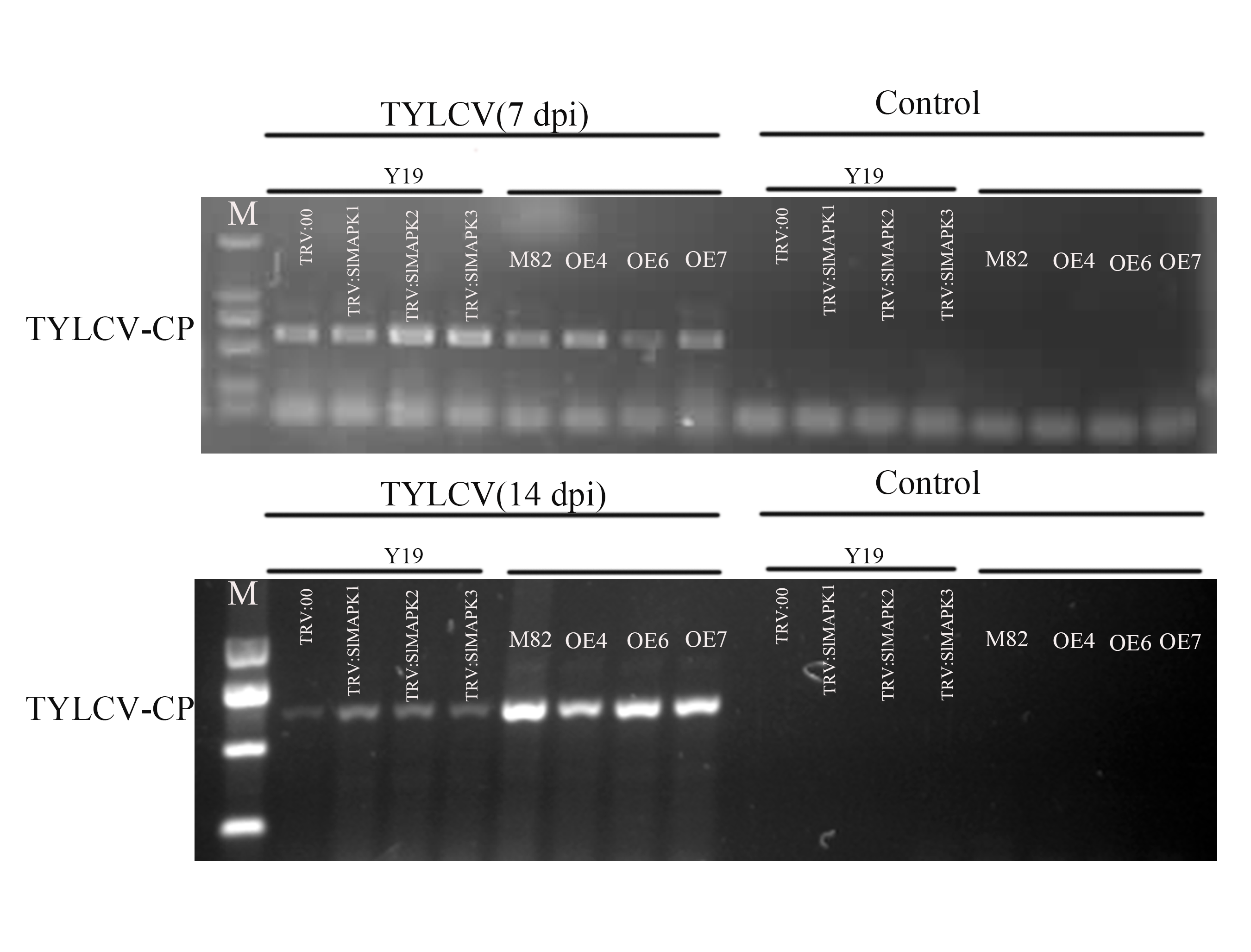

Supplement: S2 Fig — DNA was extracted from the top leaves of SlMAPK-silenced (TRV: SlMAPK1-, TRV: SlMAPK2-, and TRV: SlMAPK3-) plants, non-silenced (TRV: 00-, control) plants, ‘M82’ plants and OE plants at 7 and 14 dpi. PCR was used to confirm the success of artificial inoculation with TYLCV. (TIF) [file pone.0172466.s003.tif]

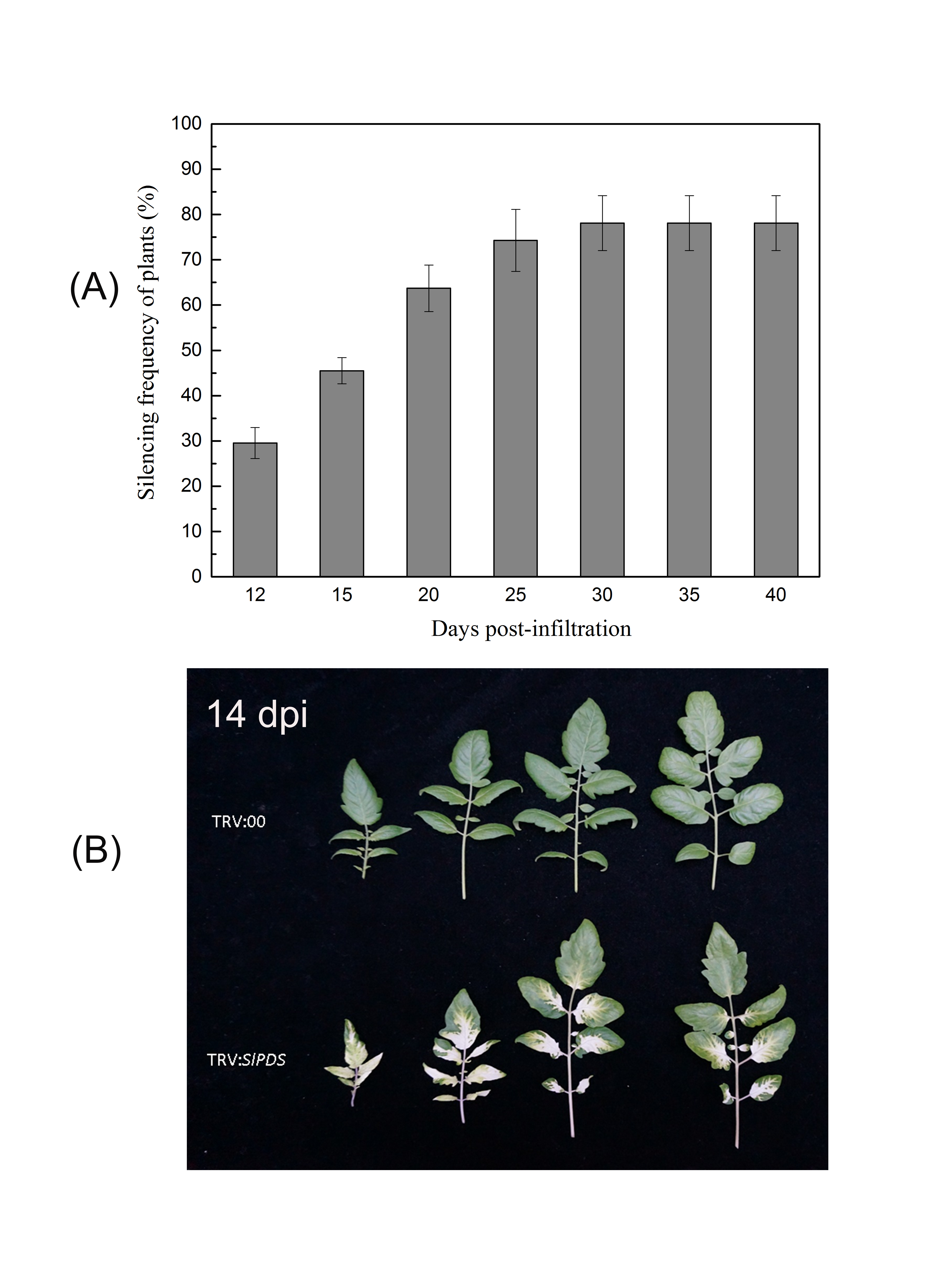

Supplement: S3 Fig — The silencing frequency (%) of VIGS technology (A) was calculated in TRV: SlPDS -infiltrated plants at 12, 15, 20, 25 30, 35 and 40 dpi. The following equation was used: Number of plants showing silencing phenotype (bleaching or yellowing)Total number of plants infiltrated×100% Plants were infected with TRV vector carrying the phytoene desaturase (PDS) gene of tomato. Silencing of the endogenous PDS results in the inhibition of carotenoid biosynthesis, leading to a photo-bleaching phenotype (B). Photographs taken 4 weeks after TRV infiltration. (TIF) [file pone.0172466.s004.tif]
